# Non-surgical Restoration of L3/L4 Disc Herniation

**DOI:** 10.7759/cureus.40941

**Published:** 2023-06-25

**Authors:** Eric Chun-Pu Chu, Edouard Sabourdy

**Affiliations:** 1 Chiropractic and Physiotherapy Centre, New York Medical Group, Hong Kong, CHN; 2 Research, Mctimoney College of Chiropractic, Abingdon, GBR; 3 Chiropractic Clinic, FV (Franco-Vietnamese) Hospital, Ho Chi Minh, VNM

**Keywords:** chiropractic, north american spine society, disc regression, lumbar disc resorption, chiropractor

## Abstract

We report the case of a 52-year-old Asian man presenting with severe lower back pain and bilateral radiculopathy due to acute L3/L4 disc extrusion, causing severe spinal canal stenosis and bilateral L4 nerve root compression. Following a ten-week conservative management plan consisting of high-velocity, low-amplitude spinal manipulation, adjunctive therapies, and home exercise, the patient experienced significant pain relief, functional improvement, and near resolution of deficits. A six-month follow-up MRI revealed the resolution of the L3/L4 disc extrusion, and the patient remained asymptomatic at nine months. This case highlights the potential for spontaneous resorption of a lumbar disc herniation and symptomatic improvement with conservative management, including chiropractic treatment. A favorable natural course of a disc herniation should be considered when deciding between early surgical intervention and conservative management, warranting further prospective studies to evaluate the effectiveness of chiropractic treatment and the role of spontaneous regression in symptomatic lumbar disc herniation.

## Introduction

Lumbar disc herniations are ubiquitous musculoskeletal conditions routinely encountered in chiropractic practice [1-5]. Although the pathophysiological mechanisms underlying nerve root compression and indications for surgical intervention are well established, the processes by which conservative modalities provide symptomatic relief and facilitate disc resorption remain unclear.

Complications can arise from overtreatment of disc herniations, which are known to often resolve spontaneously over time without therapeutic intervention. However, the natural course tends to vary and is difficult to predict accurately. The guidelines promulgated by the North American Spine Society outline the clinical assessment and management of disc herniation with lumbar radiculopathy, recommending non-operative treatment as first-line therapy [6]. Strict adherence to these guidelines can reduce the risk of overtreatment. Existing pathophysiological models that explain the manifestations of symptomatic disc disease are imperfect and frequently yield erroneous prognoses. These guidelines translate the research into reasoned contemporary recommendations for clinical practice. Strict compliance with key recommendations is fundamental for remaining up-to-date and circumventing excessive intervention.

This case report provides an instructive example of the resolution of radiculopathy and disc resorption with conservative management. The idea of avoiding overtreatment for lumbar disc herniation with radiculopathy is now widely accepted. In the face of numerous contradictory beliefs pertaining to discogenic back pain, this case report aimed to convey an evidence-informed approach to the diagnosis and management of symptomatic disc herniation.

## Case presentation

A 52-year-old Asian man presented with a one-month history of severe sharp lower back pain radiating to the posterior thighs and calves bilaterally following a volleyball game. He reported that the pain started immediately during a forceful jump and had been worsening since and rated 8/10 at the worst. The pain was aggravated by bending, sitting, and walking and was relieved only by lying supine. He had difficulty sleeping due to pain and reported disabilities in daily activities and work. He denied having any prior episodes of low back pain or sciatica. His medical and surgical histories were unremarkable.

Upon examination, his posture was antalgic during flexion. The lumbar spine range of motion (ROM) was restricted in all directions due to pain. Palpation revealed tenderness over the L3/L4 spinous processes and paraspinal musculature. Neurological testing showed a reduced right patellar reflex (0/5) and left patellar reflex (0/5). Manual muscle testing revealed bilateral L5 myotome weakness (4/5), whereas the S1 myotome was normal bilaterally (5/5). The straight leg raise was limited to 30° ROM bilaterally due to hamstring tightness and reproduced concordant pain and paresthesia radiating to the thighs and calves, being greater in the right leg than in the left. The perianal sensory loss was not observed. The results of the remaining physical examinations were normal.

**Figure 1:**
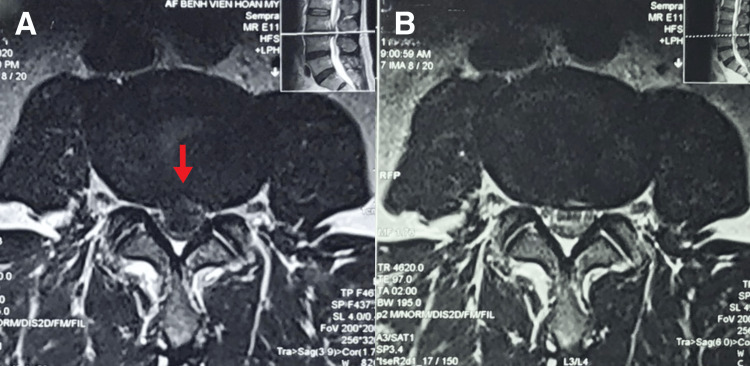
T2 MRI of the lumbar spine in axial view (A) At L3/L4, there is a severe-sized central disc extrusion (red arrow), causing severe spinal compression of the descending L4 nerve roots bilaterally within the central canal. The spinal canal shows severe narrowing, with significant central canal stenosis. (B) Nine-month follow-up examination, the image showed a total resolution of L3/L4 disc extrusion.

**Figure 2:**
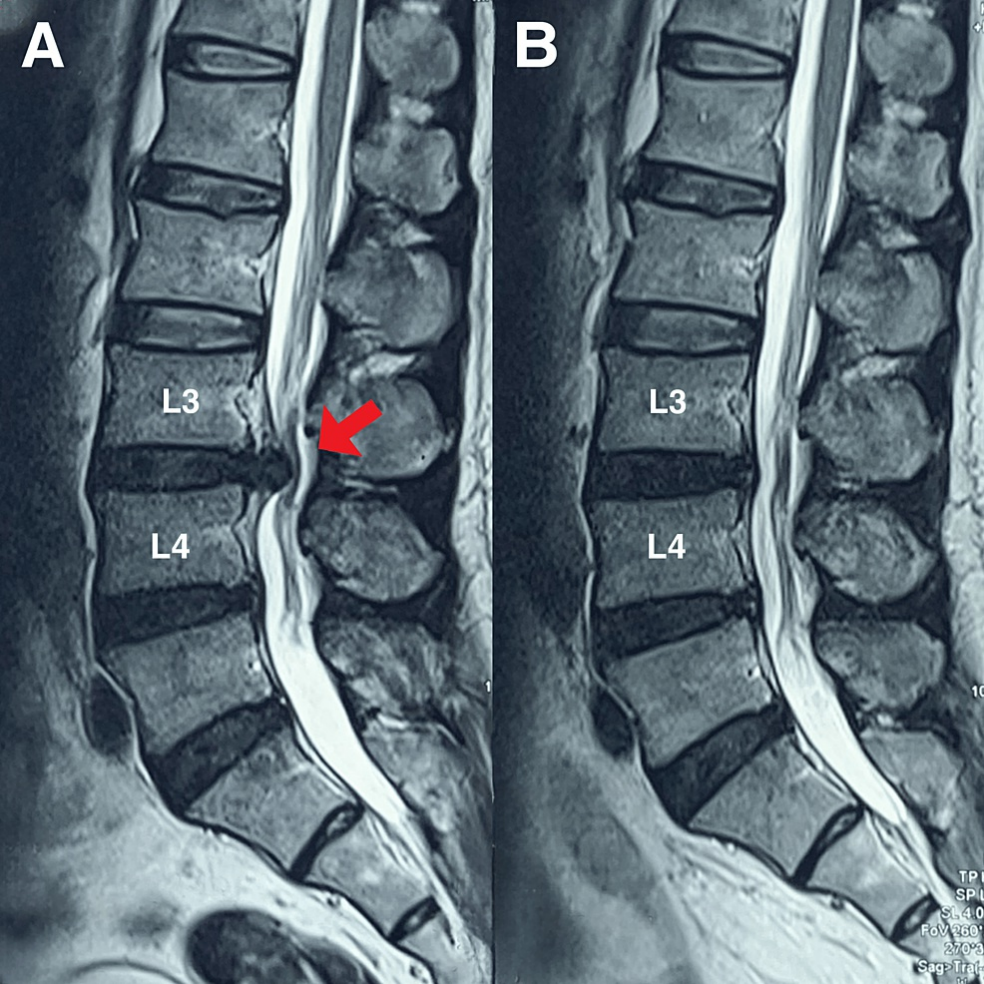
T2 MRI of the lumbar spine in sagittal view (A) The sagittal view revealed a central disc extrusion at L3/L4 measuring 12 × 8 × 6 mm (red arrow), causing severe spinal canal stenosis and compression of the descending L4 nerve roots bilaterally within the central canal. Mild disc bulges were noted at the L4/L5 and L5/S1 levels without significant stenosis. (B) A follow-up MRI of the lumbar spine revealed resolution of the L3/L4 disc extrusion.

MRI of the lumbar spine revealed a central disc extrusion at L3/L4 measuring 12 × 8 × 6 mm, causing severe spinal canal stenosis and compression of the descending L4 nerve roots bilaterally within the central canal (Figures 1A and 2A). Mild disc bulges were noted at the L4/L5 and L5/S1 levels without significant stenosis. A diagnosis of acute L3/L4 disc herniation with radiculopathy was made based on the patient’s history and clinical-radiological correlation.

The treatment plan mainly consisted of high-velocity, low-amplitude spinal manipulation of the lumbar spine, with adjunctive therapies including lumbar traction, and home exercise to assist the treatment outcome. Treatment began with daily visits per week for the initial two weeks and then reduced to two to three visits per week for the following six weeks. Significant pain relief and functional improvement were progressively achieved during each week of therapy. By the fifth week, the pain score decreased to 4/10, lumbar ROM and mobility improved by 50%, reflexes normalized, and right L5 myotome strength increased to 4+/5. The patient was able to return to work with some limitations by the sixth week. Re-evaluation at ten weeks showed near resolution of pain and deficits with mild residual tightness, at which point the frequency of care was reduced to once every one to two weeks for rehabilitation.

At six months, the patient was symptom-free, with full activity and ROM. The patient was placed on a maintenance schedule of monthly spinal manipulation to minimize recurrence. Patient perceived improvement in numeric pain scores was rated 0, and the World Health Organization quality of life questionnaire score was 95%. Nine months later, the patient remained asymptomatic. Although the patient experienced significant symptomatic improvement and functional recovery through conservative management, she expressed a strong desire for obtaining a follow-up MRI to assess the post-treatment integrity and recovery of her disc. A follow-up MRI of the lumbar spine revealed resolution of the L3/L4 disc extrusion with mild desiccation of the intervertebral disc. All other parameters were normal (Figures 1B and 2B).

## Discussion

The probability of intervertebral disc resorption in lumbar herniations remains uncertain due to the heterogeneity in imaging and morphologic factors [7]. Currently, the initial size of the herniation and sequestration are factors known to increase the likelihood of disc resorption [7]. Spontaneous resorption probabilities have been reported as 13% for bulging discs, 41% for protruding discs, 70% for extruding discs, and 96% for sequestered discs [8]. It is important to note that annular bulging is not considered a type of herniation [9]. Resorption was observed in 85.18% and 100% of cases for disc herniations with high signal intensity on T2-weighted MRI sequences and free fragments, respectively [10]. Interestingly, the more severe the herniation, the higher the chance of complete resolution, which ranges from 15% to 43% for extruded and sequestered discs, respectively [8].

Current literature reveals that symptomatic relief may not consistently coincide with a radiological improvement in disc resorption, and anatomical impairment does not always correspond with pain [8]. The cause of pain is modulated by a complex interplay between biochemical and mechanical factors [11]. Inflammatory cytokines and tumor necrosis factors, generated by immune cells, such as macrophages within degenerating discs and herniated materials, may provoke an autoimmune response at the herniation site. Axonal ischemia and nerve root pain may arise from the exposure of nerve roots to irritant substances [11-13]. Pain resulting from disc herniation predominantly originates from inflammatory responses, in addition to neurophysiological reactions and mechanical loading [14]. Excessive inflammation has also been suggested as a potential contributor to the resorption of herniated discs [14].

In this case, spinal manipulation has been observed to be a potential catalyst for this resorption process. Three hypotheses have been proposed to elucidate the common yet unpredictable phenomenon of resorption following lumbar disc herniation. The first and most extensively researched hypothesis involves a cascade of events, including inflammatory responses, macrophage infiltration, matrix remodeling, and angiogenesis [13-15]. Spinal treatment may stimulate the immune system, which recognizes herniated material as foreign, triggering macrophage phagocytosis and enzymatic degradation initiated by an inflammatory response, thereby accelerating the resorption of lumbar disc herniation. The second hypothesis posits that disc herniation size reduction occurs due to progressive dehydration and shrinkage of the nucleus pulposus, accounting for the observation that larger herniations, which possess higher water content, experience a more significant size reduction compared to smaller ones [16]. Spinal manipulation could potentially aid in this dehydration process, further contributing to the resorption of herniated discs. The third theory, mechanical retraction, proposes that herniated material formation may transpire when the pressure within the intervertebral space decreases [16], and spinal manipulation may play a role in mechanically retracting the herniated material, thus promoting resorption. Nevertheless, it is conceivable that a single mechanism or a combination of all three theories, possibly enhanced by spinal manipulation, may be responsible for the spontaneous resorption of herniated disc fragments [13].

Management principles for herniated disc treatment emphasize initial conservative approaches. Surgery should be considered a viable option only if the patient's symptoms and imaging findings do not demonstrate significant improvement following conservative treatment [17,18]. The majority of guidelines recommend that if lumbar disc herniation remains symptomatic after three months and conservative treatment proves ineffective, surgical intervention may be an option [7]. On average, 90% of patients experience improvement in radiculopathy within three months, even without surgery [19]. Current recommendations advocate for treating patients with conservative, non-operative management, including pain management, physical therapy, and activity modification, for at least six weeks before contemplating surgical intervention [6]. Surgery should be reserved for patients who fail to improve with conservative treatment or present with severe neurological complications, such as cauda equina syndrome, significant muscle weakness, or bowel or bladder dysfunction [20].

## Conclusions

In conclusion, radiculopathy brought on by disc herniation has a complex and multifactorial natural history. Disc herniation has a high probability of spontaneous resorption, particularly in severe cases. The mechanisms underlying spontaneous resorption are not well understood; however, inflammation, macrophage infiltration, matrix remodeling, and angiogenesis have been proposed as key factors. The pain associated with disc herniation arises from a combination of mechanical compression, neurophysiological changes, and inflammatory reactions. Given the favorable natural course of disc herniation, conservative management is recommended for at least six weeks before considering surgical intervention.
